# Supplementary material for: Early postnatal care contact within 24 hours by skilled providers and its determinants among home deliveries in Myanmar: Further analysis of the Myanmar Demographic and Health Survey 2015–16
Source: PLoS One. 2023 Aug 16;18(8):e0289869. doi: 10.1371/journal.pone.0289869 (PMC10431608; doi:10.1371/journal.pone.0289869)

PLOS ONE

Determinants of early postnatal care contact by skilled providers among home deliveries in Myanmar: further analysis of the Myanmar Demographic and Health Survey 2015-16

--Manuscript Draft--

**Manuscript Number:**	PONE-D-22-24579
**Article Type:**	Research Article
**Full Title:**	Determinants of early postnatal care contact by skilled providers among home deliveries in Myanmar: further analysis of the Myanmar Demographic and Health Survey 2015-16
**Short Title:**	Determinants of early postnatal contact among home deliveries in Myanmar
**Corresponding Author:**	Kyaw Lwin Show, MPH<br>Ministry of Health<br>Yangon, Yangon MYANMAR
**Keywords:**	Postnatal care; home deliveries; determinants; DHS; Myanmar
**Abstract:**	Abstract<br>Background<br>Access and use of early postnatal care (PNC) by skilled providers are crucial for reducing the maternal and newborn deaths. The magnitude and its predictors of early PNC contact by skilled providers among home deliveries in Myanmar remains unknown.<br>Methods<br>This cross-sectional study used secondary data from the Myanmar Demographic and Health Survey 2015–16. We included information on all home deliveries to interviewed mothers aged 15-49 years in the 2 years preceding the survey. Early PNC attendance and its predictors were assessed (using modified Poisson regression with robust variance estimates). All analyses were performed using STATA, Version 15. A p-value <0.05 was considered statistically significant.<br>Results<br>Of a total of 2,129 home deliveries, 36.1% (95%CI: 32.4%, 39.9%) did not receive PNC from any providers. Among all home deliveries included in this study, 468 (22.0%, 95%CI: 19.1%, 25.1%) received early postnatal checkup within 24 hours by skilled providers (doctors/nurses/midwives/Lady Health Visitors). Factors associated with early PNC contact among home deliveries by skilled providers included possessing higher education (adjusted Prevalence Ratio (aPR): 1.39, 95%CI: 1.01, 1.92), resident from coastal regions (aPR:1.40, 95%CI: 1.06, 1.84), attaining antenatal care (ANC) at least four times (aPR:1.47, 95%CI: 1.21, 1.77), giving birth by a skilled birth attendant (aPR:8.82, 95%CI: 6.68, 11.65), and having access to mass media at least once weekly (aPR:1.22, 95%CI: 1.02, 1.45).<br>Conclusion<br>A high percentage of home deliveries (78%) were not receiving early PNC by skilled providers. To facilitate early and safe PNC, expanding the coverage of skilled birth attendants and promoting the utilization of ANC should be strengthened. Targeted health education should be delivered through mass media especially for those with low education levels residing in delta, lowland, hills and plains.
**Order of Authors:**	Kyaw Lwin Show, MPH
	Pyae Linn Aung, PhD
	Thae Maung Maung, PhD
	Su Mon Myat
	Khaing Nwe Tin
**Additional Information:**	
**Question**	**Response**

Powered by Editorial Manager® and ProduXion Manager® from Aries Systems Corporation

**Financial Disclosure**<br><br>Enter a financial disclosure statement that describes the sources of funding for the work included in this submission. Review the submission guidelines for detailed requirements. View published research articles from *PLOS ONE* for specific examples.<br><br>This statement is required for submission and **will appear in the published article** if the submission is accepted. Please make sure it is accurate.<br><br>**Unfunded studies**<br>Enter: *The author(s) received no specific funding for this work.*<br><br>**Funded studies**<br>Enter a statement with the following details:<br>• Initials of the authors who received each award<br>• Grant numbers awarded to each author<br>• The full name of each funder<br>• URL of each funder website<br>• Did the sponsors or funders play any role in the study design, data collection and analysis, decision to publish, or preparation of the manuscript?<br>• **NO** - Include this sentence at the end of your statement: *The funders had no role in study design, data collection and analysis, decision to publish, or preparation of the manuscript.*<br>• **YES** - Specify the role(s) played.<br><br>* typeset	The author(s) received no specific funding for this work.
**Competing Interests**<br><br>Use the instructions below to enter a competing interest statement for this submission. On behalf of all authors, disclose any competing interests that could be perceived to bias this work—acknowledging all financial support and any other relevant financial or non-financial competing interests.<br><br>This statement is required for submission	The authors have declared that no competing interests exist.

and **will appear in the published article** if the submission is accepted. Please make sure it is accurate and that any funding sources listed in your Funding Information later in the submission form are also declared in your Financial Disclosure statement.<br><br>View published research articles from *PLOS ONE* for specific examples.<br><br>**NO authors have competing interests**<br><br>Enter: *The authors have declared that no competing interests exist.*<br><br>**Authors with competing interests**<br><br>Enter competing interest details beginning with this statement:<br><br>*I have read the journal's policy and the authors of this manuscript have the following competing interests: [insert competing interests here]*<br><br>* typeset	
**Ethics Statement**<br><br>Enter an ethics statement for this submission. This statement is required if the study involved:<br><br>• Human participants<br>• Human specimens or tissue<br>• Vertebrate animals or cephalopods<br>• Vertebrate embryos or tissues<br>• Field research<br><br>Write "N/A" if the submission does not require an ethics statement.<br><br>General guidance is provided below. Consult the submission guidelines for detailed instructions. **Make sure that all information entered here is included in the Methods section of the manuscript.**	The original DHS survey in Myanmar was conducted after obtaining approval from the Ministry of Health and ethics approval from the Ethics Review Committee on Medical Research including Human Subjects in the Department of Medical Research. The secondary data used in this study was granted by the DHS Program and the dataset provided was already de-identified.

**Format for specific study types**<br><br>**Human Subject Research (involving human participants and/or tissue)**<br>• Give the name of the institutional review board or ethics committee that approved the study<br>• Include the approval number and/or a statement indicating approval of this research<br>• Indicate the form of consent obtained (written/oral) or the reason that consent was not obtained (e.g. the data were analyzed anonymously)<br><br>**Animal Research (involving vertebrate animals, embryos or tissues)**<br>• Provide the name of the Institutional Animal Care and Use Committee (IACUC) or other relevant ethics board that reviewed the study protocol, and indicate whether they approved this research or granted a formal waiver of ethical approval<br>• Include an approval number if one was obtained<br>• If the study involved *non-human primates*, add *additional details* about animal welfare and steps taken to ameliorate suffering<br>• If anesthesia, euthanasia, or any kind of animal sacrifice is part of the study, include briefly which substances and/or methods were applied<br><br>**Field Research**<br><br>Include the following details if this study involves the collection of plant, animal, or other materials from a natural setting:<br>• Field permit number<br>• Name of the institution or relevant body that granted permission	
**Data Availability**<br><br>Authors are required to make all data underlying the findings described fully available, without restriction, and from the time of publication. PLOS allows rare exceptions to address legal and ethical concerns. See the PLOS Data Policy and FAQ for detailed information.	Yes - all data are fully available without restriction

A Data Availability Statement describing where the data can be found is required at submission. Your answers to this question constitute the Data Availability Statement and **will be published in the article**, if accepted.<br><br>**Important:** Stating 'data available on request from the author' is not sufficient. If your data are only available upon request, select 'No' for the first question and explain your exceptional situation in the text box.<br><br>Do the authors confirm that all data underlying the findings described in their manuscript are fully available without restriction?	
**Describe where the data may be found in full sentences. If you are copying our sample text, replace any instances of XXX with the appropriate details.**<br><br>• If the data are **held or will be held in a public repository**, include URLs, accession numbers or DOIs. If this information will only be available after acceptance, indicate this by ticking the box below. For example: *All XXX files are available from the XXX database (accession number(s) XXX, XXX.).*<br>• If the data are all contained **within the manuscript and/or Supporting Information files**, enter the following: *All relevant data are within the manuscript and its Supporting Information files.*<br>• If neither of these applies but you are able to provide **details of access elsewhere**, with or without limitations, please do so. For example:<br><br>*Data cannot be shared publicly because of [XXX]. Data are available from the XXX Institutional Data Access / Ethics Committee (contact via XXX) for researchers who meet the criteria for access to confidential data.*<br><br>*The data underlying the results presented in the study are available from (include the name of the third party*	We do not have the permission to share the data. However, anyone interested can access the data by making a written request to the DHS program. Further details are available in this link https://dhsprogram.com/data/available-datasets.cfm.

and contact information or URL).<br>• This text is appropriate if the data are owned by a third party and authors do not have permission to share the data.<br><br>* typeset	
Additional data availability information:	

Manuscript
Click here to access/download;Manuscript;Manuscript.docx

Determinants of early postnatal care contact by skilled providers among home deliveries in Myanmar: further analysis of the Myanmar Demographic and Health Survey 2015-16

[1]Kyaw Lwin Show*, [2]Pyae Linn Aung, [1]Thae Maung Maung, [3]Su Mon Myat, [4]Khaing Nwe Tin

[1]Department of Medical Research, Ministry of Health, Myanmar
[2]Myanmar Health Network Organization, Yangon, Myanmar
[3]Department of Public Health, Ministry of Health, Myanmar
[4]Maternal and Reproductive Division, Department of Public Health, Ministry of Health, Myanmar

*Correspondence: Kyaw Lwin Show, Email- kyawlwins@gmail.com

Abstract

Background

Access and use of early postnatal care (PNC) by skilled providers are crucial for reducing the maternal and newborn deaths. The magnitude and its predictors of early PNC contact by skilled providers among home deliveries in Myanmar remains unknown.

Methods

This cross-sectional study used secondary data from the Myanmar Demographic and Health Survey 2015–16. We included information on all home deliveries to interviewed mothers aged 15-49 years in the 2 years preceding the survey. Early PNC attendance and its predictors were assessed (using modified Poisson regression with robust variance estimates). All analyses were performed using STATA, Version 15. A p-value <0.05 was considered statistically significant.

Results

Of a total of 2,129 home deliveries, 36.1% (95%CI: 32.4%, 39.9%) did not receive PNC from any providers. Among all home deliveries included in this study, 468 (22.0%, 95%CI: 19.1%, 25.1%) received early postnatal checkup within 24 hours by skilled providers (doctors/nurses/midwives/Lady Health Visitors). Factors associated with early PNC contact among home deliveries by skilled providers included possessing higher education (adjusted Prevalence Ratio (aPR): 1.39, 95%CI: 1.01, 1.92), resident from coastal regions (aPR:1.40, 95%CI: 1.06, 1.84), attaining antenatal care (ANC) at least four times (aPR:1.47, 95%CI: 1.21, 1.77), giving birth by a skilled birth attendant (aPR:8.82, 95%CI: 6.68, 11.65), and having access to mass media at least once weekly (aPR:1.22, 95%CI: 1.02, 1.45).

Conclusion

A high percentage of home deliveries (78%) were not receiving early PNC by skilled providers. To facilitate early and safe PNC, expanding the coverage of skilled birth attendants and promoting the utilization of ANC should be strengthened. Targeted health education should be delivered through mass media especially for those with low education levels residing in delta, lowland, hills and plains.

Keywords: Postnatal care, home deliveries, determinants, DHS, Myanmar.

Background

Globally, about 295,000 maternal deaths occurred during 2017 while several efforts were made to improve maternal health. The majority of the maternal deaths (94%) occurred in low and lower to middle income countries [1]. Access to maternal health services is crucial for both mother and baby [1,2]. Most of the maternal deaths are avoidable since the major causes of maternal death are due to postpartum hemorrhage, hypertensive disorders and sepsis [2]. Furthermore, infants face a most vulnerable period for survival in their first month of birth and 2.4 million newborns dying in 2020. Most neonatal deaths happen during the first week of life and mostly due to childbirth-related complications (birth asphyxia or lack of breathing at birth), and infections [3,4]. Given the context, access and use of PNC is crucial to reduce the maternal and newborn deaths because two thirds of maternal deaths occur during the postpartum period [5].

Postnatal care (PNC) comprises that provided to women and her babies immediately after the birth until six weeks (42 days) after delivery. While the first two days after delivery present the highest risk for mother and baby, early PNC within two days after delivery is crucial for the detection of postnatal danger signs, and timely management of complications [2,6,7]. During early PNC, women can have the opportunity to discuss with healthcare providers regarding exclusive breastfeeding, and family planning. Therefore, the World Health Organization (WHO) recommends early PNC as early as possible within first 24 hours after birth for home deliveries and continue monitoring until six weeks after delivery [5].

Use of PNC among the deliveries by skilled providers in the some developing countries remains unsatisfactory. Early PNC use in Nepal, Indonesia, Ethiopia, Uganda and South Sudan totaled 19%, 78%, 34%, 50%, and 11%, respectively [8–12]. PNC was not received especially among those residing in rural community and barriers in receiving PNC included limited access to services, lack of health literacy and sociocultural beliefs [13,14].

Despite significantly improved maternal health indicators, Myanmar still has the highest MMR among ASEAN countries with 250 per 100,000 live births in 2017 and Neonatal Mortality Rate (NMR) was 22.3 per 1000 live births in 2020 [15]. The most recent Myanmar Demographic and Health Survey (MDHS) (2015-16) reported that two thirds of births in the five years preceding the survey occurred in their home and 60% of total deliveries were assisted by skilled providers [16]. Women who deliver at the healthcare institutions receive the care from the healthcare

providers since immediately after the delivery until discharge. For women who deliver at home, however, the coverage and timing of PNC is uncertain; which rely on the knowledge of women on the recommended PNC, accessibility and availability of healthcare providers.. Furthermore, the skilled of healthcare providers is crucial to obtain an effective, comprehensive and quality PNC. One study conducted in a rural area of Myanmar reported that about one third of mothers (32.6%) did not receive PNC [17]. However, literature concerning PNC among home deliveries remains limited. Therefore, this study explored the magnitude of early PNC attendance among home deliveries in Myanmar through further analysis of nationwide data to provide the inputs for reducing maternal and newborn deaths in Myanmar.

Methods

Study design and data source

We conducted a cross-sectional study using secondary data from the Myanmar Demographic and Health Survey 2015-16 (MDHS 2015-16) and included information on all home deliveries to interviewed mothers in the two years preceding the survey.

Myanmar Demographic and Health Survey 2015-16

The MDHS 2015-16 is the only nationally representative survey to collect comprehensive data concerning basic demographic, socioeconomic and health indicators of women and men aged 15 to 49 years based on the 2014 census frame. The survey followed a stratified two-stage cluster sampling design with a response rate of 98%, ensuring the representativeness of the data. The first stage involved selecting the numbers and points of clusters either a census enumeration area or ward/village tracts. A total of 442 clusters have been included independently from a total of 30 sampling strata. It has been followed by sampling a fixed number of 30 households from each cluster. All women aged 15 to 49 years in the selected households and all men aged 15 to 49 years were interviewed in every second selected household. The survey used tablet computer-assisted field editing procedures and all completed questionnaires were entered in the tablets while in the field by the field editors.

Variables

In this study, all deliveries occurred outside of healthcare institutions were considered as home deliveries. The main outcome variable in this study was ‘early postnatal care (PNC) attendance’ defined as having the first PNC checkup from a skilled provider within 24 hours after delivery. The outcome variable was coded as a binary variable; considered as ‘yes’ if women received PNC checkup from a skilled provider within 24 hours after delivery, and considered as ‘no’ if women did not receive PNC checkup within 24 hours after delivery, or women received PNC checkup from an unskilled provider. We considered doctors, nurses, midwives, and lady health visitors (LHV) as skilled providers in this study.

The independent predictor variables included mother’s completed age, place of residence, region, education level, wealth quintile, marital status, occupation, antenatal care (ANC) attendance, number of living children, exposure to mass media, attendant at birth and partner’s education. Regions were categorized according to their characteristics: delta and lowland (Ayeyawady, Yangon and Bago Regions, Mon and the Karen States), hilly (Kachin, Kayah, Chin and Shan States), coastal (Rakhine State and Tanintharyi Region) and plains (Magway, Mandalay, Sagaing Regions and Nay Pyi Taw Union Territory). Occupation was categorized as dependent (homemaker), agriculture work, manual labor, clerical/ sales/ services work and professional/ technical/ managerial work. ANC attendance was categorized as complete ANC for receiving at least four times and incomplete ANC for less than four times. Total number of living children was categorized as two or less children and more than two children. Mass media exposure was recoded as ‘yes’ when access could be gained to television, newspaper, or radio at least once weekly. Skilled attendance at birth was recoded as ‘yes’ when medical doctors, nurses or midwives/ LHV were present at birth.

Data analysis

We analyzed the data using STATA Software, Version 15 (STATA Corp., College Station, TX, USA). Background demographic data and socioeconomic status of mothers were analyzed using descriptive statistics including number, percentage, means and standard deviation. We assessed first PNC attendance within 24 hours after delivery among home deliveries using proportions and 95% CI. We used modified Poisson regression with robust variance estimates (enter method – all variables included together in a single step). We included variables with a crude p-value of <0.2 (Chi-square test) in the modified Poisson regression model. We ruled out multicollinearity among

variables (assessed using variance inflation factor) before including them in the model. We summarized the association between variables included in the model and early PNC attendance within 24 hours using Adjusted Prevalence Ratio (aPR) and 95% CI. Weight factors and the ‘svyset’ command were applied to account for the two-stage stratified cluster sampling design. A p-value of <0.05 was considered statistically significant.

Ethics consideration

The original DHS survey in Myanmar was conducted after obtaining approval from the Ministry of Health and ethics approval from the Ethics Review Committee on Medical Research including Human Subjects in the Department of Medical Research. The secondary data used in this study was granted by the DHS Program and the dataset provided was already de-identified.

Results

Background characteristics of women delivering at home

Of 2,129 women who delivered at home, more than one half were aged 25 to 34 years. Most (75.5%) attained no education or primary education level. One third (36.5%) resided in the delta and lowland regions and 89% were rural residents. Nearly two thirds (63.5%) were categorized in the two poorest quintiles. More than one half (55.4%) did not receive antenatal care (ANC) at least four times and only one third delivered their babies with a skilled birth attendant (doctors/nurses/midwives/LHV). More than one half (54.6%) had two or fewer children and 51.6% had exposure to mass media (television/newspaper/radio) at least once weekly. A total of 1,429 (67.1%) had a partner with no education or primary education level (Table 1).

Time taken to receive postnatal care

Figure 1 and table 2 describe the time taken to receive postnatal checkup by type of attendant among home deliveries. Of 2,129 home deliveries, 768 (36.1%, 95%CI: 32.4%, 39.9%) did not receive postnatal checkup and 829 (38.9%, 95%CI: 35.6%, 42.4%) received postnatal checkup within 24 hours after delivery by any type of provider.

Factors associated with early postnatal care attendance by skilled provider within 24 hours among home deliveries

Among all home deliveries, 468 (22.0%, 95%CI: 19.1%, 25.1%) received early postnatal checkup within 24 hours by skilled providers (doctors/nurses/midwives/LHV). In the bivariate analysis, women's education level (p<0.001), geographic location (p<0.001), place of residence (p<0.001), wealth (p<0.001), current marital status (p=0.013), number of ANC times received (p<0.001), number of living children (p=0.001), exposure to mass media (p<0.001), and partner's education level (p<0.001) were associated with postnatal care (PNC) attendance by skilled providers within 24 hours after delivery (Table 3).

In the multivariable analysis, women with higher education level (aPR = 1.39, 95%CI: 1.01, 1.92) were more likely to have PNC attendance by skilled providers within 24 hours after delivery compared to no education or primary education level. Women who are residing in coastal regions (aPR = 1.40, 95%CI: 1.06, 1.84) were more likely to have PNC attendance by skilled providers within 24 hours after delivery compared to those residing in hilly regions. Women with ANC taken at least 4 times (aPR = 1.47, 95%CI: 1.21, 1.77), delivered by skilled providers (aPR = 8.82, 95%CI: 6.68, 11.65), and exposed to mass media (aPR = 1.22, 95%CI: 1.02, 1.45) were more likely to receive postnatal checkup by skilled providers within 24 hours after delivery (Table 3).

Table 1. Background characteristics of women who delivered at home included in the Myanmar Demographic and Health Survey (n=2129).

**Characteristics**	**Frequency**	**Percentage**
Completed age		
15-24 years	397	18.7
25-34 years	1077	50.5
35-49 years	655	30.8
Education		
No education/Primary	1607	75.4
Secondary	472	22.2
Higher	50	2.4
Region		
Delta and lowland	778	36.5
Hills	451	21.2
Coastal	241	11.3
Plains	659	31.0
Residence		
Rural	1903	89.4

Urban	227	10.6
Wealth index		
Poorest	789	37.1
Poorer	563	26.4
Middle	380	17.8
Richer	310	14.6
Richest	88	4.1
Current marital status		
Married	2027	95.2
Widowed/Divorced/Separated	102	4.8
Occupation		
Dependent / Home maker	705	33.2
Agriculture	391	18.4
Manual labor	694	32.6
Clerical/sales/services	281	13.2
Professional/technical/managerial	55	2.6
Antenatal care taken		
At least 4 times	950	44.6
Less than 4 times	1179	55.4
Birth attendant		
Doctor	25	1.2
Nurse/midwife/lady health visitor	753	35.4
Auxiliary midwife	198	9.3
Traditional birth attendant	923	43.3
Relative/friend	205	9.6
No one	16	0.7
Others	10	0.5
Number of living children		
Two or less children	1163	54.6
More than two children	966	45.4
Mass media exposure		
At least once a week	1098	51.6
Less than once a week	1031	48.4
Partner's education		
No education/ Primary	1429	67.1
Secondary	628	29.5
Higher	27	1.3
Don't know	45	2.1

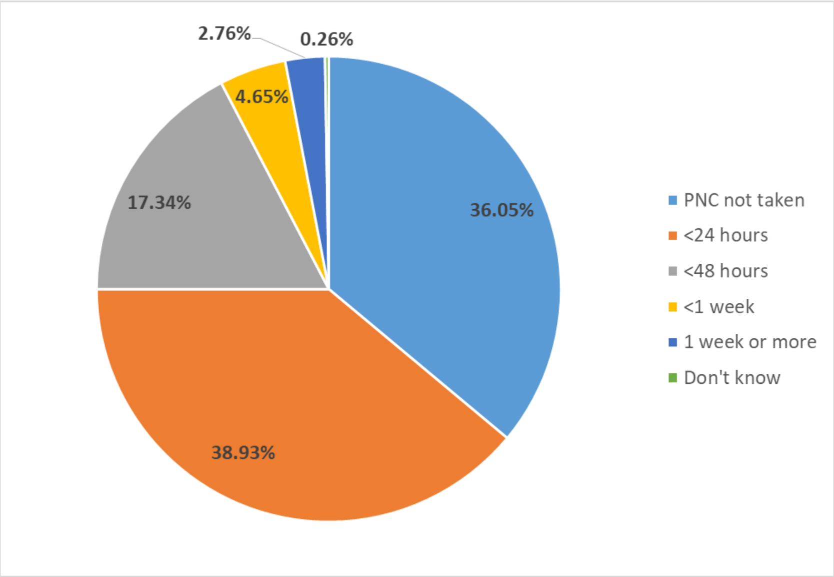

Figure 1. Time taken to receive postnatal care contact among home deliveries by any type of providers in the Myanmar Demographic and Health Survey (n=2129).

Table 2. Postnatal care contact by attendant among home deliveries in the Myanmar Demographic and Health Survey (n=1361).

**Provider**	**Within 24 hours**		**1-2 days**		**>2 days**	
	**n**	**%**	**n**	**%**	**n**	**%**
Doctor	18	2.2	5	1.3	4	2.4
Nurse/midwife/LHV	450	54.2	182	49.3	96	58.9
Auxiliary midwife	99	11.9	36	9.7	20	12.3
Traditional birth attendant	247	29.8	138	37.5	42	25.8
Community/village health worker	5	0.6	7	2.0	0	0
Others	11	1.3	1	0.2	1	0.6
**Total**	**829**	**100**	**369**	**100**	**163**	**100**

LHV: lady health visitor.

Table 3. Factors associated with early postnatal care contact by skilled provider within 24 hours among home deliveries in the Myanmar Demographic and Health Survey (n=2129).

**Characteristics**	**Total**	**PNC within 24 hours by skilled provider**					
	**N**	**n**	**%**	**cPR**	**p value**	**aPR**	**95% CI**
Total	2129	468	22.0				
Completed age					0.407		
15-24 years	397	81	20.5	Ref:			
25-34 years	1077	229	21.3	1.04			
35-49 years	655	157	24.0	1.17			
Education					<0.001		
No education/Primary	1607	298	18.6	Ref:		Ref:	
Secondary	472	141	29.9	1.61		0.95	0.79, 1.15
Higher	50	28	56.3	3.03		1.39	**1.01, 1.92**
Region					<0.001		
Delta and lowland	778	159	20.5	1.20		1.05	0.83, 1.32
Hills	451	77	17.1	Ref:		Ref:	
Coastal	241	38	15.7	0.92		1.40	**1.06, 1.84**
Plains	659	193	29.3	1.71		1.12	0.90, 1.41
Residence					<0.001		
Rural	1903	390	20.5	Ref:		Ref:	
Urban	227	78	34.3	1.68		0.98	0.79, 1.22
Wealth index					<0.001		
Poorest	789	107	13.6	Ref:		Ref:	
Poorer	563	109	19.3	1.42		1.07	0.85, 1.36
Middle	380	100	26.2	1.93		1.16	0.91, 1.48
Richer	310	107	34.3	2.53		1.18	0.91, 1.55
Richest	88	46	52.5	3.87		1.17	0.84, 1.61
Current marital status					0.013		
Married	2027	457	22.6	2.19		1.79	0.97, 3.32
Widowed/Divorced/Separated	102	10	10.3	Ref:		Ref:	
Occupation					0.064		

Dependent / Home maker	705	143	20.4	Ref:		Ref:	
Agriculture	391	76	19.5	0.96		1.12	0.88, 1.43
Manual labor	694	152	21.9	1.08		1.05	0.86, 1.27
Clerical/sales/services	281	80	28.4	1.40		0.96	0.76, 1.21
Professional/technical/managerial	55	16	29.3	1.44		0.85	0.57, 1.25
Antenatal care taken					<0.001		
At least 4 times	950	320	33.7	2.69		1.47	**1.21, 1.77**
Less than 4 times	1179	148	12.5	Ref:		Ref:	
Birth attendant					<0.001		
Skilled birth attendant	778	402	51.8	10.75		8.82	**6.68, 11.65**
Unskilled birth attendant	1351	65	4.8	Ref:		Ref:	
Number of living children					0.001		
Two or less children	1163	290	25.0	1.36		1.04	0.88, 1.23
More than two children	966	177	28.3	Ref:		Ref:	
Mass media exposure					<0.001		
At least once a week	1098	311	28.3	1.86		1.22	**1.02, 1.45**
Less than once a week	1031	157	15.2	Ref:		Ref:	
Partner's education					<0.001		
No education/ Primary	1429	254	17.8	Ref:		Ref:	
Secondary	628	190	30.3	1.71		1.06	0.90, 1.26
Higher	27	15	54.8	3.09		1.01	0.70, 1.44

PNC: postnatal care; cPR: Crude Prevalence Ratio; aPR: Adjusted Prevalence Ratio; CI: confidence interval.

Discussion

This constitutes the first report documenting the early postnatal care (PNC) contact by skilled providers among home deliveries using the country's representative samples in Myanmar. The results estimated relatively poor early PNC contact (22% in this study) by skill providers within home deliveries. Among institutional deliveries in Myanmar, majority (93%) took PNC and nearly 80% received PNC checkup within 24 hours after discharge [16]. A study conducted in rural Myanmar reported that full PNC services use among home deliveries was 14.6% [17]. The difference in use rate might be due to different outcome definitions; that study investigated full PNC services contact (receiving at least four postnatal visits and the first visit within 24 hours of delivery) and our study identified early PNC use within 24 hours after delivery. Furthermore, this study focused on both rural and urban home deliveries in Myanmar. The findings are more or less similar to studies reporting PNC use within 48 hours in Zambia (17.6% in 2014) and Ethiopia (34% in 2019) and lower than studies conducted in Indonesia (87% in 2017), Bangladesh (52% in 2017) and the Philippines (86% in 2017) [18–22]. The variation in presenting this use rate might be due to different operational definitions for outcome variables and differences in access to service and health systems by different countries. The potential reasons for low use of PNC are due to lack of knowledge of pregnancy-related complications or difficulty in accessing and reaching care [23].

The attainment of a higher level of education was significantly associated with early PNC contact in the current study. The finding was consistent with other studies conducted in Pakistan, Bangladesh and Nepal [24–26]. Women with higher education attainment are more likely to obtain knowledgeable maternal health information including health risks and access to care from the reliable sources of information and have a better understanding of it.

This study observed a higher chance of PNC use among women residing in coastal regions (Rakhine State and Tanintharyi Region) than those living in hilly terrain (Kachin, Kayah, Chin and Shan States). This can be explained by the fact that the hilly regions experience difficulty to access to healthcare services because of limited transportation capacities. The finding was consistent with studies conducted in Pakistan and Nepal [24,26]. This finding suggests possible alternatives for delivering PNC services especially to those residing in remote areas with difficult transportation for both mothers as well as healthcare providers.

The current study showed that those predisposed to antenatal care (ANC) at least four times as WHO recommends have a higher opportunity of using early PNC. The finding is similar to results of studies conducted in Nepal, rural India and Ethiopia [23,24,27]. Women were usually more familiar with receiving ANC services and creating the opportunity to build trust between healthcare professionals and women. Furthermore, healthcare professionals can inform the importance of using PNC services, their availability and recommended timing and frequency of postnatal visits.

Delivery attended by skilled providers is crucial for both mothers and babies; it has been recognized as a lifesaving mechanism and remains the key strategy for averting MMR and NMR. Difficulty in accessing to skilled birth attendants and community acceptance of unskilled providers (traditional birth attendants) were the main reasons for not being delivered by skilled providers in Myanmar [28]. In this study, women delivering using skilled providers were more likely to utilize early PNC, which was similar to findings from Indonesia, Nepal, Zambia and India [21,24,27,29]. Community health worker/voluntary health worker (VHW) plays vital role in continuum of care, educating women about the importance of PNC for both mother and baby, and communication between providers and women. VHW should be strengthened in term of capacity building and communication especially in resource limited countries.

Health information disseminated through mass media such as listening to the radio, watching television, or reading newspapers could improve women's ability to seek appropriate health services in a timely manner [30]. Furthermore, limited access to mass media would make it difficult for women to reach essential health knowledge and information regarding the importance of early PNC. In this study, women who exposed to mass media at least once weekly experienced a higher chance of using early PNC checkup, which was consistent with findings of studies conducted in Indonesia, Bangladesh and Nigeria [29,31,32].

This study encountered a number of strengths. This constitutes the first report accessing early use of PNC within 24 hours among home deliveries in Myanmar as recommended by the WHO postnatal guidelines. The findings are nationally representative and based on a robust dataset (double data entry and validation). Nonetheless, the study encountered a few limitations. First, due to the cross-sectional nature of the data, temporality between potential predictors and early use of PNC cannot be assessed. Second, due to the nature of secondary data analysis, some potential

predictors including knowledge and attitude towards early PNC contact and reasons for not using PNC services remained unexplored. Despite limitations, this study provides essential information to improve maternal and newborn services aiming to reduce morbidity and mortality of mother and newborn in Myanmar.

Conclusion

Despite early PNC being essential to avoid unnecessary maternal and neonatal deaths, in this study, a considerably high percentage of home deliveries (78%) lacked receiving PNC by skilled health providers within 24 hours postpartum period. Therefore, to facilitate early and safe PNC, expanding the coverage of skilled birth attendants and promoting the utilization of ANC should be strengthened. Targeted health education regarding importance of early PNC should be delivered through mass media especially for those with low education levels residing in delta, lowland, hills and plains regions. A qualitative study exploring the detailed difficulties and challenges hindering the use of early PNC among home deliveries is recommended.

Acknowledgments

We would like to thank the Demographic and Health Surveys (DHS) Program for granting access to the survey datasets.

Funding

The authors received no specific funding for this work.

Data availability statement

We do not have the permission to share the data. However, anyone interested can access the data by making a written request to the DHS program. Further details are available in this link https://dhsprogram.com/data/available-datasets.cfm.

Competing interests

The authors have declared that no competing interests exist.

Author contributions

KLS was the principal investigator. KLS, PLA, TMM, and KNT involved in conception and designing the study. KLS analyzed the data. All authors involved in providing critical review to the manuscript, and approval of the manuscript from this protocol.

References

[1] Trends in Maternal Mortality: 1990 to 2015: Estimates by WHO, UNICEF, UNFPA, World Bank Group and the United Nations Population Division. 2019.

[2] Say L, Chou D, Gemmill A, Tunçalp Ö, Moller A-B, Daniels J, et al. Global causes of maternal death: a WHO systematic analysis. Lancet Glob Heal 2014;2:e323–33. doi:10.1016/S2214-109X(14)70227-X.

[3] World Health Organization. WHO recommendations on maternal and newborn care for a positive postnatal experience. 2022.

[4] World Health Organization. Newborn Mortality 2022. https://www.who.int/news-room/fact-sheets/detail/levels-and-trends-in-child-mortality-report-2021 (accessed September 3, 2022).

[5] World Health Organization (WHO). Postnatal care of the mother and newborn. 2013.

[6] Barnett S, Nair N, Tripathy P, Borghi J, Rath S, Costello A. A prospective key informant surveillance system to measure maternal mortality - findings from indigenous populations in Jharkhand and Orissa, India. BMC Pregnancy Childbirth 2008;8. doi:10.1186/1471-2393-8-6.

[7] Maswime S, Buchmann E. Causes and avoidable factors in maternal death due to cesarean-related hemorrhage in South Africa. Int J Gynecol Obstet 2016;134:320–3. doi:10.1016/J.IJGO.2016.03.013.

[8] Dhakal S, Chapman GN, Simkhada PP, van Teijlingen ER, Stephens J, Raja AE. Utilisation of postnatal care among rural women in Nepal. BMC Pregnancy Childbirth 2007;7:19. doi:10.1186/1471-2393-7-19.

[9] Sebayang SK, Has EMM, Hadisuyatmana S, Efendi F, Astutik E, Kuswanto H. Utilization of Postnatal Care Service in Indonesia and its Association with Women's Empowerment: An Analysis of 2017 Indonesian Demographic Health Survey Data. Matern Child Health J 2022;26:545–55. doi:10.1007/S10995-021-03324-Y/TABLES/2.

[10] Gebrehiwot TG, Mekonen HH, Gebru TH, Kiros KG, Gebresilassie B, Teklu G, et al. <p>Prevalence and Associated Factors of Early Postnatal Care Service Use Among Mothers Who Had Given Birth Within the Last 12 Months in Adigrat Town, Tigray, Northern Ethiopia, 2018</p>. Int J Womens Health 2020;12:869–79. doi:10.2147/IJWH.S266248.

[11] Ndugga P, Namiyonga NK, Sebuwufu D ogratious. Determinants of early postnatal care attendance: analysis of the 2016 Uganda demographic and health survey. BMC Pregnancy Childbirth 2020;20:1–14. doi:10.1186/S12884-020-02866-3/TABLES/3.

[12] Izudi J, Akwang GD, Amongin D. Early postnatal care use by postpartum mothers in Mundri East County, South Sudan. BMC Health Serv Res 2017;17. doi:10.1186/S12913-017-2402-1.

[13] Probandari A, Arcita A, Kothijah K, Pamungkasari EP. Barriers to utilization of postnatal care at village level in Klaten district, central Java Province, Indonesia. BMC Health Serv Res 2017;17:1–9. doi:10.1186/S12913-017-2490-Y/TABLES/2.

[14] Balde MD, Diallo A, Soumah AM, Sall AO, Diallo BA, Barry F, et al. Barriers to Utilization of Postnatal Care: A Qualitative Study in Guinea. Open J Obstet Gynecol 2021;11:391–402. doi:10.4236/OJOG.2021.114039.

[15] World Health Organization. Trends in Maternal mortality ratio (modeled estimate, per 100,000 live births) - Myanmar, Lao PDR, Singapore, Bangladesh, Thailand, Cambodia, Philippines, Indonesia, Brunei Darussalam, Vietnam | 2000-2017: Estimates by WHO, UNICEF, UNFPA, World Bank Group 2019. https://data.worldbank.org/indicator/SH.STA.MMRT?locations=MM-LA-SG-BD-TH-KH-PH-ID-BN-VN (accessed May 15, 2021).

[16] Ministry of Health and Sports (MoHS) and ICF. Myanmar Demographic and Health

Survey 2015-16. Nay Pyi Taw, Myanmar, and Rockville, Maryland USA: 2017.

[17] Mon AS, Phyu MK, Thinkhamrop W, Thinkhamrop B. Utilization of full postnatal care services among rural Myanmar women and its determinants: a cross-sectional study. F1000Research 2018;7. doi:10.12688/F1000RESEARCH.15561.1.

[18] Ethiopia Mini Demographic and Health Survey 2019: Final Report. Rockville, Maryland, USA: EPHI and ICF: 2021.

[19] Indonesia Demographic and Health Survey 2017. Jarkarta, Indonesia: BKKBN, BPS, Kemenkes, and ICF: 2018.

[20] Bangladesh Demographic and Health Survey 2017-18. Dhaka, Bangladesh, and Rockville, Maryland, USA: NIPORT and ICF: 2020.

[21] Chungu C, Makasa M, Chola M, Jacobs CN. Place of Delivery Associated With Postnatal Care Utilization Among Childbearing Women in Zambia. Front Public Heal 2018;6:94. doi:10.3389/FPUBH.2018.00094/BIBTEX.

[22] Philippines National Demographic and Health Survey 2017. Quezon City, Philippine, and Rockville, Maryland, USA: PSA and ICF: 2018.

[23] Tesfahun F, Worku W, Mazengiya F, Kifle M. Knowledge, Perception and Utilization of Postnatal Care of Mothers in Gondar Zuria District, Ethiopia: A Cross-Sectional Study. Matern Child Health J 2014;18:2341. doi:10.1007/S10995-014-1474-3.

[24] Khanal V, Adhikari M, Karkee R, Gavidia T. Factors associated with the utilisation of postnatal care services among the mothers of Nepal: analysis of Nepal Demographic and Health Survey 2011. BMC Womens Health 2014;14:19. doi:10.1186/1472-6874-14-19.

[25] Mosiur Rahman M, Haque SE, Sarwar Zahan M. Factors affecting the utilisation of postpartum care among young mothers in Bangladesh. Health Soc Care Community 2011;19:138–47. doi:10.1111/J.1365-2524.2010.00953.X.

[26] Saira A, Wilson LA, Ezeh KO, Lim D, Osuagwu UL, Agho KE. Factors associated with non-utilization of postnatal care among newborns in the first 2 days after birth in Pakistan: a nationwide cross-sectional study. Glob Health Action 2021;14.

doi:10.1080/16549716.2021.1973714/SUPPL_FILE/ZGHA_A_1973714_SM7013.DOCX.

[27] Singh PK, Rai RK, Alagarajan M, Singh L. Determinants of Maternity Care Services Utilization among Married Adolescents in Rural India. PLoS One 2012;7. doi:10.1371/JOURNAL.PONE.0031666.

[28] Oo K, Win L Le, Saw S, Mon MM, Oo YTN, Maung TM, et al. Challenges faced by skilled birth attendants in providing antenatal and intrapartum care in selected rural areas of Myanmar. WHO South-East Asia J Public Heal 2012;1:467. doi:10.4103/2224-3151.207049.

[29] Titaley CR, Dibley MJ, Roberts CL. Factors associated with non-utilisation of postnatal care services in Indonesia. J Epidemiol Community Heal 2009;63:827–31. doi:10.1136/JECH.2008.081604.

[30] Ware H. Effects of maternal education, women's roles, and child care on child mortality. Child Surviv Strateg Res 1984:191–214. doi:10.2307/2807961.

[31] Islam MR, Odland JO. Determinants of antenatal and postnatal care visits among indigenous people in Bangladesh: A study of the Mru community. Rural Remote Health 2011;11:1–13. doi:10.22605/rrh1672.

[32] Agho KE, Ezeh OK, Issaka AI, Enoma AI, Baines S, Renzaho AMN. Population attributable risk estimates for factors associated with non-use of postnatal care services among women in Nigeria. BMJ Open 2016;6:e010493. doi:10.1136/BMJOPEN-2015-010493.

Figure

Click here to access/download;Figure;Figure1.png

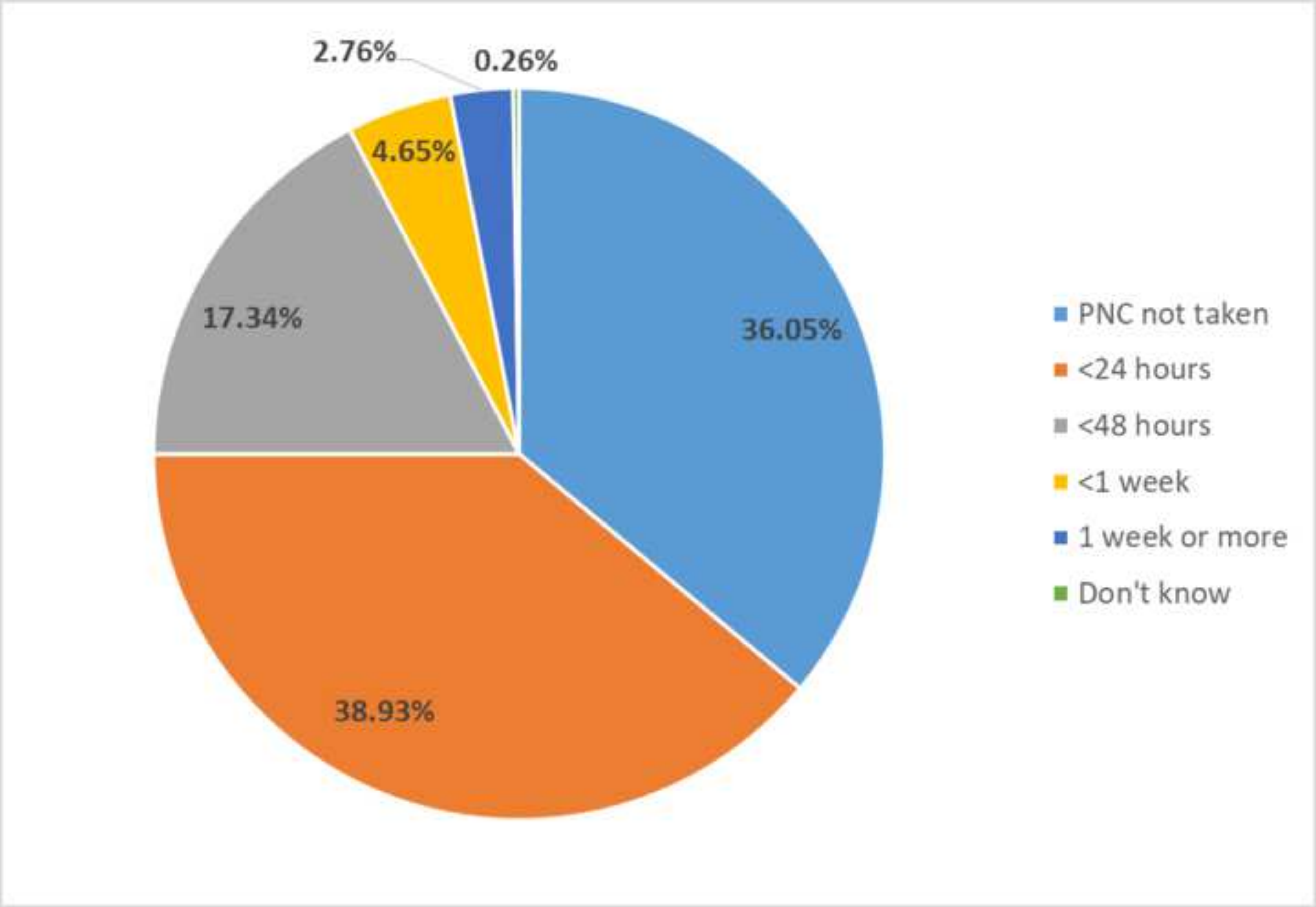

Supplement: Attachment — Submitted filename: Manuscript_Commented.pdf [file pone.0289869.s002.pdf]
