# Supplementary material for: Early postnatal care contact within 24 hours by skilled providers and its determinants among home deliveries in Myanmar: Further analysis of the Myanmar Demographic and Health Survey 2015–16
Source: PLoS One. 2023 Aug 16;18(8):e0289869. doi: 10.1371/journal.pone.0289869 (PMC10431608; doi:10.1371/journal.pone.0289869)
Supplement: Attachment — Submitted filename: Comments to the editor & the authors for PLOS ONE.docx [file pone.0289869.s001.docx]

**Comments to the editor**

Thank you for the opportunity to review this article on ‘Determinants of early postnatal care contact by skilled providers among home Deliveries in Myanmar’

- The gab need to be filled was not stated in the abstract section
- The background section is bulky with unnecessary details
- The study was conducted with no objective.
- There are variables need operational definition, but not operationalized
- Ethical consideration was not written with reference number
- This study was conducted with several unclear points in the manuscript and the text should be checked carefully for mistakes, typos.
- Some paragraphs were not cited.

Generally the result finding is nearly good and different from published articles in different journals on the same title, so in my opinion, publishing this article has significance for readers if the mentioned comments are corrected.

**Comments to the authors**

First**,** I would like to appreciate your effort to contribute your finding

**General comments**

- This study needs revision because of there are several unclear points in the manuscript and the text should be checked carefully for mistakes and typos.
- The study was conducted with no objective.
- The background section is bulky with unnecessary details.

**Specific comments**

I found that the study is interesting; however, there are many problems in the manuscript. Specific comments are listed below.

**Abstract section**

- The gab need to be filled was not stated. So, you are expected summarize the gabs you are filled by conducting this study

**Background**

- I like the way you wrote your background part, but haven’t got strong justification why this study is needed to be conducted and its significance.
- The paragraph below is not cited under the introduction section (Line 67 to 71); it needs revision.

*“Women who deliver at the healthcare institutions receive the care from the healthcare providers since immediately after the delivery until discharge. For women who deliver at home, however, the coverage and timing of PNC is uncertain; which rely on the knowledge of women on the recommended PNC, accessibility and availability of healthcare providers. Furthermore, the skilled of healthcare providers is crucial to obtain an effective, comprehensive and quality PNC”*

- As you know Research objective is a statement that clearly depicts the goal to be achieved. Concerning this I have tried to search to see the objective of this study, but I didn’t found it. Generally the research is conducted without objective.

**Methods**

- I appreciate that you defined some of the variables. The operational definition that you incorporate is less effect on outcome variables so it is better to operationalize Auxiliary midwife and Traditional birth attendant
- Considering ethical issue is good; So, reference number must be written

**Result**

*Line 153 and 154 stated that, “Among all home deliveries, 468 (22.0%, 95%CI: 19.1%, 25.1%) received early postnatal checkup within 24 hours by skilled providers (doctors/nurses/midwives/LHV).” But, under variables (line 94 and 95), you narrate as “all deliveries occurred outside of healthcare institutions were considered as home deliveries. The main outcome variable in this study was ‘early postnatal care (PNC) attendance’ defined as having the first PNC checkup from a skilled provider within 24 hours after delivery.“* I am confusing with this two controversial statements. Thus, it needs your justification.

**Discussion**

- The discussion needs more explanation based on result findings and reference it appropriately.
