# Supplementary material for: Early postnatal care contact within 24 hours by skilled providers and its determinants among home deliveries in Myanmar: Further analysis of the Myanmar Demographic and Health Survey 2015–16
Source: PLoS One. 2023 Aug 16;18(8):e0289869. doi: 10.1371/journal.pone.0289869 (PMC10431608; doi:10.1371/journal.pone.0289869)
Supplement: Attachment — Submitted filename: Responses to reviewers.docx [file pone.0289869.s003.docx]

**Dear Editors and Reviewers.**

Thank you for the detailed constructive comments, critiques, and suggestions. We have now attempted to address each point and provide a point-by-point response in below. Page and Line numbers are referred to clean manuscript file.

Thank you for your consideration and look forward to hearing from you.

Sincerely,

Dr. Kyaw Lwin Show, on behalf of the authors

**REVIEWER:**

**Reviewer #1**:

**Comments to the authors**

First**,** I would like to appreciate your effort to contribute your finding

**General comments**

- This study needs revision because of there are several unclear points in the manuscript and the text should be checked carefully for mistakes and typos.

**RESPONSE:**

We thoroughly reviewed the grammar and spelling in the manuscript using Grammarly software and received assistance from a native scientific writer for English language editing.

**REVIEWER:**

- The study was conducted with no objective.

**RESPONSE:**

We have added an objective statement in the final part of the Background section. (LINE 15 and	LINE 77)

**REVIEWER:**

- The background section is bulky with unnecessary details.

**RESPONSE:**

We now revised the Background section and removed some unnecessary sentences.

**REVIEWER:**

**Specific comments**

I found that the study is interesting; however, there are many problems in the manuscript. Specific comments are listed below.

**Abstract section**

- The gab need to be filled was not stated. So, you are expected summarize the gabs you are filled by conducting this study

**RESPONSE:**

We now added the research gab in the Abstract (LINE 14).

**REVIEWER:**

**Background**

- I like the way you wrote your background part, but haven’t got strong justification why this study is needed to be conducted and its significance.

**RESPONSE:**

We have added additional information to strengthen the justification for the study. We added “However, literature concerning PNC among home deliveries remains limited. To date, there is no documented evidence in Myanmar that has explored the utilization of PNC services and its associated factors among home deliveries. Therefore, this study aims to investigate the extent of PNC contact especially within 24 hours by skilled providers and the factors related to it among home deliveries in Myanmar. The results of this study will furnish crucial insights that can be utilized to decrease morbidity and mortality of mothers and newborns in Myanmar”. (LINE 75)

**REVIEWER:**

- The paragraph below is not cited under the introduction section (Line 67 to 71); it needs revision.

*“Women who deliver at the healthcare institutions receive the care from the healthcare providers since immediately after the delivery until discharge. For women who deliver at home, however, the coverage and timing of PNC is uncertain; which rely on the knowledge of women on the recommended PNC, accessibility and availability of healthcare providers. Furthermore, the skilled of healthcare providers is crucial to obtain an effective, comprehensive and quality PNC”*

**RESPONSE:**

We added citation to the information. (LINE 72)

**REVIEWER:**

- As you know Research objective is a statement that clearly depicts the goal to be achieved. Concerning this I have tried to search to see the objective of this study, but I didn’t found it. Generally the research is conducted without objective.

**RESPONSE:**

We now added the objective statement to the last part of the Background section. We added as “Therefore, this study aimed to investigate the extent of PNC contact especially within 24 hours by skilled providers and the factors related to it among home deliveries in Myanmar. The results of this study will furnish crucial insights that can be utilized to decrease morbidity and mortality of mothers and newborns in Myanmar”. (LINE 77)

**REVIEWER:**

**Methods**

- I appreciate that you defined some of the variables. The operational definition that you incorporate is less effect on outcome variables so it is better to operationalize Auxiliary midwife and Traditional birth attendant

**RESPONSE:**

Thank you. We mentioned as “We considered doctors, nurses, midwives, and lady health	visitors (LHV) as skilled providers in this study” (LINE 118). All other providers were	considered as unskilled providers in this study. We hope this is acceptable.

**REVIEWER:**

- Considering ethical issue is good; So, reference number must be written

**RESPONSE:**

The original MDHS study was reviewed and approved by Ethics Review Committee, Department of Medical Research from Myanmar and ICF Institutional Review Board, and the data is fully de-identified and anonymized. Furthermore, the data is publicly available at the DHS program website <<https://dhsprogram.com/data/available-datasets.cfm>> and the authorization letter to use the survey datasets has been granted by the DHS program officials. The present study has comprised a secondary data analysis of its data and ethics approval was not required.

**REVIEWER:**

**Result**

*Line 153 and 154 stated that, “Among all home deliveries, 468 (22.0%, 95%CI: 19.1%, 25.1%) received early postnatal checkup within 24 hours by skilled providers (doctors/nurses/midwives/LHV).” But, under variables (line 94 and 95), you narrate as “all deliveries occurred outside of healthcare institutions were considered as home deliveries. The main outcome variable in this study was ‘early postnatal care (PNC) attendance’ defined as having the first PNC checkup from a skilled provider within 24 hours after delivery.“* I am confusing with this two controversial statements. Thus, it needs your justification.

**RESPONSE:**

Thank you. The main outcome in our study was early PNC within 24 hours by skilled	providers (doctors/nurses/midwives/lady health visitors) among home deliveries in	Myanmar. We have made it clearer in the manuscript.

**REVIEWER:**

**Discussion**

- The discussion needs more explanation based on result findings and reference it appropriately.

**RESPONSE:**

We have revised the discussion part as needed.

**REVIEWER:**

**Reviewer #2**:

Thank you very much for an opportunity to review this article. I have presented my comments about the paper as follows. Additionally, you have comments within the electronic copy of your manuscript uploaded with this message.

General:

- There are many serious grammatical, punctuation and other language errors which make understanding of your paper difficult. Please thoroughly go through the manuscript and brush up all these language errors. I strongly advise you to seek help of someone who has very good knowledge of English and has experience of writing scientific paper.

**RESPONSE:**

We thoroughly reviewed the grammar and spelling in the manuscript using Grammarly software and received assistance from a native scientific writer for English language editing.

**REVIEWER:**

Title:

- Your title says “Determinants of early postnatal contact…”, whereas the internal content of the paper attempts to discuss both proportion of early PNC contact and factors associated with it. So, either title or internal content of the paper should be amended to remove this inconsistency.

**RESPONSE:**

We now changed the title to “Early postnatal care contact within 24 hours by skilled	providers and its predictors among home deliveries in Myanmar: further analysis of the	Myanmar Demographic and Health Survey 2015-16”.

**REVIEWER:**

Abstract:

- Objective/aim of the study is not clearly mentioned. Objective/aim of the study should be mention in the first paragraph of the abstract.

**RESPONSE:**

The objective of the study is now included in the first paragraph of the abstract. (LINE 15)

**REVIEWER:**

- Methods: clearly highlight how predictors of early PNC were assessed.

**RESPONSE:**

Thank you.

**REVIEWER:**

Background:

- Ideas are not appropriately glued to each other. So please do the followings: A) Re-arrange your paragraphs logically so that ideas flow smoothly. B) Present only one main idea and its supporting statements within a single paragraph. C) Within a single paragraph, appropriately develop and support the main idea.

**RESPONSE:**

Thank you. We have revised the Background part.

**REVIEWER:**

- In this part of your paper, you put much focus on maternal and child health problems with little emphasis on early PNC (i.e main focus of your study). It is excellent to highlight epidemiology of maternal and child health problems, but you should majorly deal with PNC issues (what is, why, how, what is known about its effectiveness, etc). Particularly make sure that the last 3-4 paragraphs discuss early PNC sufficiently and then conclude by justifying the important of the study.

**RESPONSE:**

We now revised with more focus on early PNC.

**REVIEWER:**

- The research gap which motivated you to do this study is not clearly shown except few statements. Please clearly indicate the research gap which motivated you to undertake this study with appropriate reference/s.

**RESPONSE:**

We have added additional information to strengthen the justification for the study. (LINE 75)

**REVIEWER:**

- Why you specifically motivated to study predictors of early PNC contact only among women who delivered at home? Why not all women? Please present justification for this.

**RESPONSE:**

We included as “Women who deliver at the healthcare institutions receive care from healthcare providers immediately after delivery until discharge, but for women who deliver at home, the coverage and timing of PNC is uncertain relying on the women’s knowledge of recommended PNC, accessibility and availability of healthcare providers.”. (LINE 69)

**REVIEWER:**

Methods:

- Your research cannot be replicated- Because your methodology is not presented clearly and with sufficient detail. Your methods and materials section should be written again so that it is clear and detailed enough.

- Sufficient description of study setting (i.e Myanmar) is needed.

**RESPONSE:**

Thank you. We now added study setting in the Methods part. (LINE 85)

**REVIEWER:**

- Detailed description of the followings is essential: sample size, sampling procedures, study population, from where you got data and how data extraction was done.

**RESPONSE:**

We have added further information to the Methodology section. However, as the data used in this study is publicly available survey data, we were unable to include certain information, such as sample size considerations. We hope this is acceptable.

**REVIEWER:**

Results:

- I think you have to work further to better refine titles of your sub-headings. Some of them do not make full sense. E.g- “Time taken to receive postnatal care”-what does it mean? Is it time to get the service? Or Time to reach to where PNC is provided? Please re-examine all your sub-headings.

**RESPONSE:**

Thank you. We have revised as “Utilization of postnatal care contact by any type of provider among home deliveries”. (LINE 168)

**REVIEWER:**

- Line 147-151: Some of information presented under subheading “Time taken to receive postnatal care” are not related to the subheading. E.g- proportion of women who received PNC. Normally, the contents should be reflected in the title of the subheading.

**RESPONSE:**

Thank you. We have revised as “Utilization of postnatal care contact by any type of provider among home deliveries”. (LINE 168)

**REVIEWER:**

- Why you specifically interested to present the “Time taken to receive postnatal care” as a separate title in your results section?

**RESPONSE:**

We have revised it to "Utilization of postnatal care contact by any type of provider among home deliveries". Although our primary outcome is early PNC within 24 hours post-delivery by a skilled provider, we also wish to report on the overall utilization of PNC in Myanmar, as we believe this information will be useful for the reader.

**REVIEWER:**

- Line 149-155: you presented proportion of women who received PNC, but under two different subheadings. Related concepts should be presented and discussed under the same subheading in a connected manner to enable ease understanding.

**RESPONSE:**

We have revised it to "Utilization of postnatal care contact by any type of provider among home deliveries". Although our primary outcome is early PNC within 24 hours post-delivery by a skilled provider, we also wish to report on the overall utilization of PNC in Myanmar, as we believe this information will be useful for the reader.

**REVIEWER:**

- Table 1 and Figure 1 should be put immediately after paragraph/s describing them.

**RESPONSE:**

We now moved the Table and Figure immediately after the text describing them.

**REVIEWER:**

Discussion:

- The discussion lacks focus and is very superficial. Your discussion should be focused on early postnatal care/contact. Also compare and contrast your findings with existing science/literatures as much as possible and, then show how they relate to what is known, as well what they imply.

**RESPONSE:**

We have revised the discussion part.
