# Supplementary material for: Early postnatal care contact within 24 hours by skilled providers and its determinants among home deliveries in Myanmar: Further analysis of the Myanmar Demographic and Health Survey 2015–16
Source: PLoS One. 2023 Aug 16;18(8):e0289869. doi: 10.1371/journal.pone.0289869 (PMC10431608; doi:10.1371/journal.pone.0289869)
Supplement: Attachment — Submitted filename: Responses to reviewers.docx [file pone.0289869.s004.docx]

**Ref: PONE-D-22-24579**

Early postnatal care contact within 24 hours by skilled providers and its predictors among home deliveries in Myanmar: further analysis of the Myanmar Demographic and Health Survey 2015-16

**Dear Editors and Reviewers.**

Thank you for the comments. We have addressed the editor’s and reviewer’s comments and responded point by point below. We hope the revised manuscript now meets your and the reviewer’s expectations. We have also taken this opportunity to made editorial changes to improve clarity and readability of the manuscript.

We are submitting a revised manuscript with track changes as well as a clean revised manuscript. The line numbers referred to by us in the response to reviewer’s comments (below) refers to the line numbers in the cleaned manuscript.

Thank you for your consideration and look forward to hearing from you.

Sincerely,

Dr. Kyaw Lwin Show, on behalf of the authors

**EDITOR:**

Thanks for your work. Here are my comments:

1. Abstract Line 15: "Identify the magnitude of early PNC contact" --> estimate the prevalence of having early PNC Contact...

**RESPONSE:**

Thank you. We have revised it as advised. (Page 2, Line 17)

2. Introduction: Please add previous studies on PNC utilization in low- and middle-income countries and mention previous findings of these studies.

**RESPONSE:**

Thank you. We now included the findings from previous studies in low- and middle-income countries. We included as “However, use of PNC among the deliveries by skilled providers in the some developing countries remains unsatisfactory. Wealth, mother’s education, uptake of antenatal care, place of delivery and access to media were associated with uptake of PNC.” (Page 4, Line 62-65)

3. Methods Line 90: "We conducted a cross-sectional study using secondary data from the.." --> We conducted a secondary analysis of the...

**RESPONSE:**

Thank you. We have revised it as advised. (Page 5, Line 95)

4. Methods: What is the basis for the variables you selected in the study? Please cite previous studies that would justify the inclusion (or exclusion) of certain variables)

**RESPONSE:**

Thank you. We now explained it in the Methods part and cite the references. (Page 7,	Line 146)

5. Methods: You only included mothers who have given birth at home, which is fine. Can you have a section on your study population, how you actually chose them, and how did you ensure that the standard errors remain ok even if you only studied a particular subpopulation?

**RESPONSE:**

Thank you very much for this comment. As we used the secondary data, we have to specify our study population within the available dataset. However, DHS used weights to adjust for the underrepresentation. Furthermore, in our study, we used the modified Poisson regression with robust variance estimates to provide unbiased estimates. We hope this is fine.

6. Methods: What's the basis for the p<0.2 cutoff? Any theoretical basis for your variable selection strategy?

**RESPONSE:**

Thank you very much for this comment. We wanted to explore the possible predictors of early PNC contact in Myanmar. In our study, we included variables if their unadjusted p value was less than p<0.2. This was to ensure that the model was parsimonious and also prevented over-adjustment. As we were exploring the predictors for early PNC contact it was difficult to assess potential confounders for each and every variable and then include them in the model.

7. Methods: Since you used Poisson regression, have you assessed for overdispersion? Can you describe the methodology that you have used and its results?

**RESPONSE:**

Thank you very much for this comment. We tested goodness-of-fit test and resulted p value >0.05. Therefore, we can say that the model fits reasonably well. We have clarified this in line 149 of revised manuscript.

8. Results: Clarify which of the results are weighted or not. Please indicate which analyses are weighted and which are not.

**RESPONSE:**

Thank you. All analyses were weighted to account for two-stage stratified cluster sampling design. We have clarified this in line 151 of revised manuscript and also in the tables’ footnotes.

9. Results: When you say it is adjusted PR, which variables have you adjusted for? Please indicate as a footnote.

**RESPONSE:**

Thank you. We now included as footnote. (Table 3)

10. Results: Where is the adjusted PR for age? Please control for age even if it is not significant in the crude as it is theoretically a known confounder in most associations.

**RESPONSE:**

Thank you very much for this comment. We understand ‘Age’ as universal confounder. However, in our study, we consider that every woman who delivered a baby should have early PNC irrespective of her age. Furthermore, to avoid for over-adjustment, we would like to rule out ‘Age’ in our final model. We hope this is fine.

11. Conclusion Line 283: Remove "period".

**RESPONSE:**

Thank you. We have revised it as advised. (Page 17, Line 326)

**REVIEWER:**

**Reviewer #1**:

First, I would like to appreciate your commitment to consider the comments given.

Specific comments

I found that the study is interesting. Despite, many problems in the manuscript were revised; still some issues need to be addressed as listed below.

Abstract section

• On the background of the abstract section it is good to add the extent of the burden of early PNC across the globe.

**RESPONSE:**

Thank you. We now added information regarding unsatisfactory PNC in developing countries in the background of the abstract section. (Page 2, Line 14)

**REVIEWER:**

• Is that predictors and associated factors are similar or can we use them interchangeability? Because on the topic predictor is used, but in the objective section associated factor is used. It has to be consistent.

**RESPONSE:**

Thank you. We have revised it as advised and make it consistent.

**REVIEWER:**

• Under the keyword, the word determinant is non-significant for the study since the title is modified and again it needs revision.

**RESPONSE:**

Thank you. We have revised it as advised. (Page 3, Line 41)

**REVIEWER:**

• The text should be checked carefully for mistakes, typos and grammar throughout the whole manuscript.

**RESPONSE:**

Thank you. We thoroughly reviewed the grammar and spelling in the manuscript using Grammarly software and received assistance from a native scientific writer for English language editing.

**REVIEWER:**

References

• References like, reference number 2, 6, 8,21,27,28,30,31,32 and 34 are outdated. Thus, the references used must be the recent one.

**RESPONSE:**

Thank you. We have made the necessary updates to the citation and references, incorporating the most recent information available. However, we would like to keep certain references that support our information and lack of updated data.

**REVIEWER:**

**Reviewer #3**:

First of all, I would like to appreciate all of your team’s effort on this study.

General comment

This study needs more and deep additional discussion points. It is recommended to revise and do proofreading systematically.

**RESPONSE:**

Thank you. We now discussed deeper by adding additional discussion points.

**REVIEWER:**

Specific comments

Abstract

Adding the research gap in this section is incomplete. It would be good to describe why your topic/study is very important for Myanmar.

**RESPONSE:**

Thank you. We now added the research gap for Myanmar in the abstract. (Page 2, Line 15)

**REVIEWER:**

Background

The way of writing in the background section is acceptable. It would be good to include more information about the problem and justification of the study, as there are some interesting issue than limited literature.

**RESPONSE:**

Thank you for the comment. We now added more to make our study more justifiable.

**REVIEWER:**

Method

It is good that you explained about the operational definition of variables in this section. It is needed to express how to extract the data/sample (step by step) from the data set. I am aware of you used the secondary data. However, I hope you could explain how to get the final sample because the whole sample included the woman who did not mention their delivery place. How did you treat those kind of women in your study?

**RESPONSE:**

Thank you for the comment. In our study, we considered as ‘home deliveries’ if the delivery occurred outside of the healthcare institutions. WHO recommends early PNC as early as possible within the first 24 hours after birth for those who delivered outside healthcare institution. We mentioned it as “In this study, all deliveries occurred outside of healthcare institutions were considered as home deliveries.” (Page 6, Line 117)

**REVIEWER:**

Presenting the final sample size before and after weighting is excellent.

**RESPONSE:**

Thank you. We now included both weighted and unweighted results in Table 1.

**REVIEWER:**

Results

It would be nice to present the unweighted and weighted results side by side in Table 1.

**RESPONSE:**

Thank you. We now included both weighted and unweighted results in Table 1.

**REVIEWER:**

Discussion

The discussion section needs more and deep explanation. A comparison and contrast of existing literature from Myanmar and other South East Asia countries are necessary for each variable to be discussed. Although limited, there are some literature on PNC in Myanmar. It could be strengthened by addressing on some of the initiatives taken by the government to improve access to maternal health services and reflected the current situation of health care workforce, health infrastructures and security concern in Myanmar.

**RESPONSE:**

Thank you for the comment. We now discussed deeper by adding additional discussion points.

**REVIEWER:**

References

The reference format should be consistent. The reference style of numbers 22, 23, 24 and 26 are different from others. You can reflect by reviewing the reference number 16.

**RESPONSE:**

Thank you. We have revised accordingly. We used Mendeley referencing software and followed ‘PLOS ONE’ referencing style.
