# Supplementary material for: Early postnatal care contact within 24 hours by skilled providers and its determinants among home deliveries in Myanmar: Further analysis of the Myanmar Demographic and Health Survey 2015–16
Source: PLoS One. 2023 Aug 16;18(8):e0289869. doi: 10.1371/journal.pone.0289869 (PMC10431608; doi:10.1371/journal.pone.0289869)
Supplement: Attachment — Submitted filename: Comments to the editor & the authors for PLOS ONE.docx [file pone.0289869.s005.docx]

**Comments to the editor**

Thank you for the more opportunity given to me to review this manuscript again ‘**early postnatal care contact within 24 hours by skilled providers and its predictors among 2 home deliveries in Myanmar: further analysis of the Myanmar Demographic and Health 3 Survey 2015-16**’

- The background section is bulky with unnecessary details and the author need to be summarized
- Ethical consideration was not written with its ethics review board containing reference number
- This study was conducted with several unclear points in the manuscript and the text should be checked carefully for mistakes, typos.

Generally the result finding is nearly good and different from published articles in different journals on the same title, so in my opinion, publishing this article has significance.

**Comments to the authors**

It is good to appreciate you for coming up with the modified manuscript based on the given comments and suggestion.

**General comments**

- This study needs revision because of there are several unclear points in the manuscript and the text should be checked carefully for mistakes and typos.
- Still the background section is bulky with unnecessary details even though it was commented previously.
- The ethics approval from the Ethics Review Committee on Medical Research is not addressed with its reference number. Dear author, please, get back to your ethical consideration and incorporate it
