# Supplementary material for: Early postnatal care contact within 24 hours by skilled providers and its determinants among home deliveries in Myanmar: Further analysis of the Myanmar Demographic and Health Survey 2015–16
Source: PLoS One. 2023 Aug 16;18(8):e0289869. doi: 10.1371/journal.pone.0289869 (PMC10431608; doi:10.1371/journal.pone.0289869)
Supplement: Attachment — Submitted filename: Responses to reviewers.docx [file pone.0289869.s006.docx]

**Ref: PONE-D-22-24579**

Early postnatal care contact within 24 hours by skilled providers and its determinants among home deliveries in Myanmar: further analysis of the Myanmar Demographic and Health Survey 2015-16

**Dear Editors and Reviewers.**

Thank you for the comments. We have addressed the editor’s and reviewer’s comments and responded point by point below. We hope the revised manuscript now meets your and the reviewer’s expectations.

We are submitting a revised manuscript with track changes as well as a clean revised manuscript. The line numbers referred to by us in the response to reviewer’s comments (below) refers to the line numbers in the cleaned manuscript.

Thank you for your consideration and look forward to hearing from you.

Sincerely,

Dr. Kyaw Lwin Show, on behalf of the authors

**EDITOR:**

Additional Editor Comments:

Dear authors, thanks for your work. I have already received two accept reviews and am close to accepting your paper, but I still have issues on your manuscript that I would like you to address.

1. Change predictors --> determinants. Your modelling strategy is not looking for predictors.

**RESPONSE:**

Thank you very much. We now changed predictors to determinants throughout the manuscript.

2. I have mentioned this previously, but I want a separate section on your study population. In your abstract you mentioned that you only included information on all home deliveries to interviewed mothers aged 15-49 in the 2 years preceding the survey. How did you do this with your dataset? What functionalities/Stata commands have you used to ensure that the standard errors are still appropriate?

**RESPONSE:**

Thank you very much. We now included a separate section on study population. (LINE 116). To account for the complex survey design, we used the commend ‘svyset’ in our analyses to ensure robust standard errors that account for the complex sampling structure. This ensures that the estimated standard errors accurately represent the variability in the population. Furthermore, we considered robust variance estimates to obtain robust standard errors for the parameter estimates as recommended by Cameron and Trivedi (2009).

3. It is entirely possible that a mother could be counted twice because she can give birth twice in the 2 years preceding the survey. This will cause duplications on her age and other socio-demographic characteristics of the mother. How did you address this issue? Put this under the Study Population section that I am suggesting.

**RESPONSE:**

Thank you very much for this very important point and sorry for the confusion. Actually, we only included information regarding the last delivery. We now mentioned this in study population section. (LINE 117). We filtered the study population to last delivery by the STATA command ‘keep if bidx==1’.

4. What specific dataset recode did you use?

**RESPONSE:**

We used the BR file from the DHS dataset and now mentioned in the study population section. (LINE 117)

5. The Hosmer Lemeshow goodness of fit test is used for logistic models. You are using Poisson models so you need to find a goodness of fit test appropriate for Poisson models, but more than goodness of fit, I am more interested in your assessment of potential overdispersion and how you handled it if its present.

6. I have raised this previously but how did you assess for potential overdispersion? This should be checked because you are using a Poisson model which is very sensitive to overdispersion.

**RESPONSE:**

Thank you very much. We are not aware of any command to perform model-fitness test for modified poisson regression in STATA. We are aware of model fitness test using the poisson command (estat gof). We repeated the model using this command and the model-fitness test resulted p value >0.05.

Regarding the overdispersion, we assessed using the Pearson and deviance statistics. We have checked the values of the Pearson Chi Squared statistic/Degrees of freedom and the Deviance Statistics/Degrees of freedom and both values are <1.

7. I have raised this previously, but I want you to control for age. Previous studies have found that age is really a determinant of PNC utilization. <https://www.ncbi.nlm.nih.gov/pmc/articles/PMC8140851/>

**RESPONSE:**

We now controlled for the variable ‘Age’ in our adjusted model. (Table 3)

8. Please address the remaining comments of the reviewers.

**RESPONSE:**

Thank you. We have also addressed the reviewers’ comments as well.

**REVIEWER:**

**Reviewer #1**:

Reviewer #1: It is good to appreciate you for coming up with the modified manuscript based on the given comments and suggestion.

General comments

o This study needs revision because of there are several unclear points in the manuscript and the text should be checked carefully for mistakes and typos.

**RESPONSE:**

Typos are checked throughout the manuscript.

**REVIEWER:**

o Still the background section is bulky with unnecessary details even though it was commented previously.

**RESPONSE:**

Thank you very much. In order to provide a comprehensive understanding of the topic for readers who may not be familiar with it, we would like to keep as it is without removing the information. We hope this is fine.

**REVIEWER:**

• The ethics approval from the Ethics Review Committee on Medical Research is not addressed with its reference number. Dear author, please, get back to your ethical consideration and incorporate it

**RESPONSE:**

The original MDHS was approved by Ethics Review Committee on Medical Research of Myanmar. The present study used the publicly available secondary data which can be downloaded from the DHS program website < <https://dhsprogram.com/>>. The dataset was already de-identified and the authorization to use the survey datasets has been granted by the DHS program officials.

**REVIEWER:**

**Reviewer #3**:

Reviewer #3: I would like to appreciate all of your team’s effort on the revision for this study. Your team addressed almost all of the comments except the reference section.

Reference number 22,23,24 are needed to be revised. For example, the recommended citation for reference number 23 is "National Population and Family Planning Board (BKKBN), Statistics Indonesia (BPS), Ministry of Health (Kemenkes), and ICF. 2018. Indonesia Demographic and Health Survey 2017. Jakarta, Indonesia: BKKBN, BPS, Kemenkes,and ICF." For this, your team need to write according to the journal (PLOS ONE)'s acceptable citation style.

**RESPONSE:**

Thank you. We have revised it.
